# Blood RNA Sequencing Indicates Upregulated *BATF2* and *LY6E* and Downregulated *ISG15* and *MT2A* Expression in Children with Autism Spectrum Disorder

**DOI:** 10.3390/ijms23179843

**Published:** 2022-08-30

**Authors:** Irena Voinsky, Yazeed Zoabi, Noam Shomron, Moria Harel, Hanoch Cassuto, Joseph Tam, Shannon Rose, Adrienne C. Scheck, Mohammad A. Karim, Richard E. Frye, Adi Aran, David Gurwitz

**Affiliations:** 1 Department of Human Molecular Genetics and Biochemistry, Faculty of Medicine, Tel Aviv University, Tel Aviv 69978, Israel; 2 Department of Cell and Developmental Biology, Faculty of Medicine, Tel Aviv University, Tel Aviv 69978, Israel; 3 Edmond J. Safra Center for Bioinformatics, Tel Aviv University, Tel Aviv 69978, Israel; 4 Sagol School of Neuroscience, Tel Aviv University, Tel Aviv 69978, Israel; 5 Shaare Zedek Medical Center, Jerusalem 91031, Israel; 6 Obesity and Metabolism Laboratory, Institute for Drug Research, School of Pharmacy, Faculty of Medicine, The Hebrew University of Jerusalem, Jerusalem 91120, Israel; 7 Department of Pediatrics, University of Arkansas for Medical Sciences and Arkansas Children’s Research Institute, Little Rock, AR 72205, USA; 8 Barrow Neurological Institute, Phoenix Children’s Hospital, Phoenix, AZ 85016, USA; 9 Department of Child Health, University of Arizona College of Medicine-Phoenix, Phoenix, AZ 85004, USA; 10 Rossignol Medical Center, Phoenix, AZ 85050, USA

**Keywords:** autism spectrum disorder (ASD), RNA sequencing, real-time qPCR, cancer

## Abstract

Mutations in over 100 genes are implicated in autism spectrum disorder (ASD). DNA SNPs, CNVs, and epigenomic modifications also contribute to ASD. Transcriptomics analysis of blood samples may offer clues for pathways dysregulated in ASD. To expand and validate published findings of RNA-sequencing (RNA-seq) studies, we performed RNA-seq of whole blood samples from an Israeli discovery cohort of eight children with ASD compared with nine age- and sex-matched neurotypical children. This revealed 10 genes with differential expression. Using quantitative real-time PCR, we compared RNAs from whole blood samples of 73 Israeli and American children with ASD and 26 matched neurotypical children for the 10 dysregulated genes detected by RNA-seq. This revealed higher expression levels of the pro-inflammatory transcripts *BATF2* and *LY6E* and lower expression levels of the anti-inflammatory transcripts *ISG15* and *MT2A* in the ASD compared to neurotypical children. *BATF2* was recently reported as upregulated in blood samples of Japanese adults with ASD. Our findings support an involvement of these genes in ASD phenotypes, independent of age and ethnicity. Upregulation of *BATF2* and downregulation of *ISG15* and *MT2A* were reported to reduce cancer risk. Implications of the dysregulated genes for pro-inflammatory phenotypes, immunity, and cancer risk in ASD are discussed.

## 1. Introduction

Autism spectrum disorder (ASD) is the most complex human neurological disorder. Inherited or de novo mutations in over 100 known genes are already implicated in ASD, yet in most incidences, the etiology remains unexplained. ASD may result from detrimental epigenetic modifications during early embryonic development [1,2]. Further contributors to the highly diverse ASD phenotypes include unique combinations of common variants in many genes (a high polygenic risk score); prenatal environmental influences [3]; mitochondrial deficiencies [4,5]; a chronic pro-inflammatory state [6,7]; psychiatric and neurologic comorbidities [8]; and aberrant gut microbiome composition [9]. Gene expression is controlled by an epigenetic mechanism including differential gene methylation [10]. Although such epigenetic changes can be inherited, other environmental factors, such as prenatal vitamin intake and prenatal tobacco exposure, can modify methylation, resulting in long-term changes in environmentally induced gene expression changes [11]. Together, the diverse phenotypes contribute to difficulties in the early diagnosis of ASD among children, which is crucial for early treatment and parental guidance [12,13]. 

The involvement of epigenetics in ASD phenotypes hinders efforts to establish improved diagnostic tools for early identification of ASD and its sub-phenotypes using genomic, biochemical, and metabolomics analyses [14]. Brain imaging diagnostic tools such as fMRI have also been suggested; however, such tools still have low stratification value and low reproducibility and thus require further studies before their incorporation into routine pediatric neurology practice [5,15,16]. Genome-wide transcriptomic studies examine changes in gene expression that also reflect epigenetic DNA modifications, thereby circumventing complex and costly technologies such as DNA methylome profiling [17]. Indeed, since the completion of the human genome project, numerous studies have applied genome-wide transcriptomics, initially using microarrays and more recently using RNA-sequencing (RNA-seq) technologies, for identifying altered gene expression patterns in blood samples from individuals with ASD compared to neurotypical (NT) controls. 

Venous blood samples represent an accessible, affordable, and readily available biological resource for establishing differential diagnosis, prognosis, and subtyping of complex disorders such as ASD. Indeed, the majority of transcriptomic studies in humans have utilized venous blood samples. Hence, we aimed to perform comparative RNA-seq in an independent cohort of whole blood samples from children with ASD and a neurotypical control group to identify the top dysregulated gene transcripts and to assess (by literature survey) whether some of the detected dysregulated genes were already reported as dysregulated in ASD. Here, we report that our comparative RNA-seq identified two upregulated mRNA transcripts, *BATF2* (basic leucine zipper ATF-like transcription factor 2) and *LY6E* (lymphocyte antigen 6 family member E), and two downregulated genes, *ISG15* (ISG15 ubiquitin-like modifier) and *MT2A* (metallothionein 2A) in whole blood samples of 73 Israeli and American children with ASD compared to 26 NT children. 

## 2. Results

### 2.1. RNA Sequencing and Real-Time qPCR Validation 

**Figure 1:**
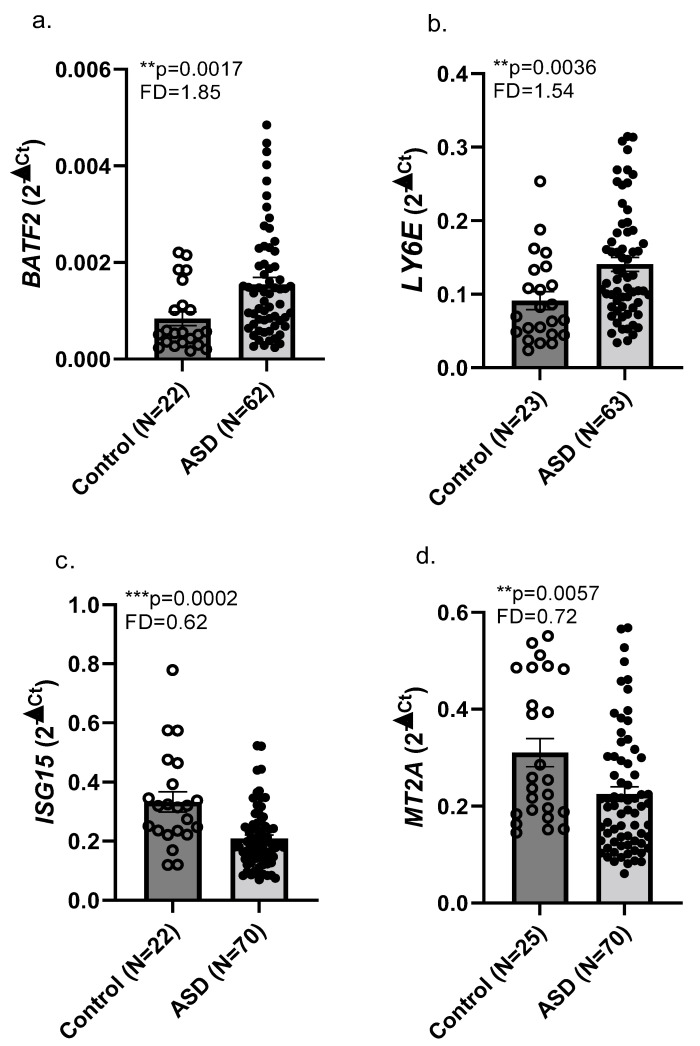
Real-time qPCR validation for RNA expression levels in Israeli and American whole blood samples from ASD and control children. Box plots show mRNA levels in whole blood samples for ASD vs. neurotypical control (mean ± SEM) for the following genes: (**a**) *BATF2*; (**b**) *LY6E*; (**c**) *ISG15*; (**d**) *MT2A*. Outliers were removed, and analysis was conducted using a non-parametric Mann–Whitney test. FD and *p* values are shown for the genes with differential expression in ASD vs. neurotypical controls. FD, fold difference (ASD vs. controls). ** *p* < 0.01; *** *p* < 0.001.

Whole blood samples were collected from 73 children with ASD and 26 NT children at Shaare Zedek Medical Center (Jerusalem, Israel) and Phoenix Children’s Hospital (Phoenix, AZ, USA). The demographics of the Israeli and U.S. cohorts are presented in Table 1. RNA was extracted from these blood samples as described in Methods. RNA from eight males with ASD (mean age 13.5 ± 2.6 y) and nine NT males (controls; mean age 15.4 ± 1.9 y) were used for RNA-seq analyses as described in the Methods. Bioinformatic analysis of the RNA-seq reads, followed by *p*-value adjustment for genome-wide transcriptomics, identified 10 dysregulated genes with differential expression in whole blood in the ASD compared to the control group, with p_adj_ < 0.05 (Table 2). Next, we performed real-time qPCR validation for these dysregulated genes in both the Israeli and American cohorts of whole blood samples from ASD and matched NT children; Table 1). Our real-time qPCR findings showed that four of the genes detected as dysregulated by RNA-seq were dysregulated in the combined Israeli and American cohorts of whole blood RNA samples (Figure 1). We observed upregulated expression of *BATF2* and *LY6E* (FD = 1.85, *p* = 0.0017; FD = 1.54, *p* = 0.0036) and downregulated expression of *ISG15* and *MT2A* (FD = 0.62, *p* = 0.0002; FD = 0.72, *p* = 0.0057) in the ASD compared to the NT control children. Other genes found as dysregulated in RNA-seq analysis of our discovery cohort blood samples (Table 2) were not confirmed in the combined cohorts. Real-time qPCR findings from the Israeli cohort alone agreed with our findings of upregulated *BATF2* and downregulated *ISG15* and *MT2A* in the combined Israeli and American children with ASD compared with NT children and, in addition, showed upregulated *FCGR1A* in the Israeli children with ASD (Supplementary Figure S1).

We looked for correlations between dysregulated mRNA levels in whole blood with behavioral phenotypes of the children with ASD. In the Israeli cohort, we observed weak negative correlations (R < 0.5) between whole blood mRNA levels of BATF2 or SERPING1 and their individual teacher SRS (tSRS) scores. Weak positive correlations (R < 0.5) were also observed in the same cohort for mRNA levels of LY6E with VABS socialization domain scores and VABS composite scores and for mRNA levels of ISG15 with CBCL scores (Supplementary Figure S2). No correlations of behavioral scores with mRNA levels with *p* < 0.05 were observed for the children with ASD in the American cohort.

### 2.2. Correlations of Whole Blood Gene Expression Levels with Serum Endocannabinoids

**Figure 2:**
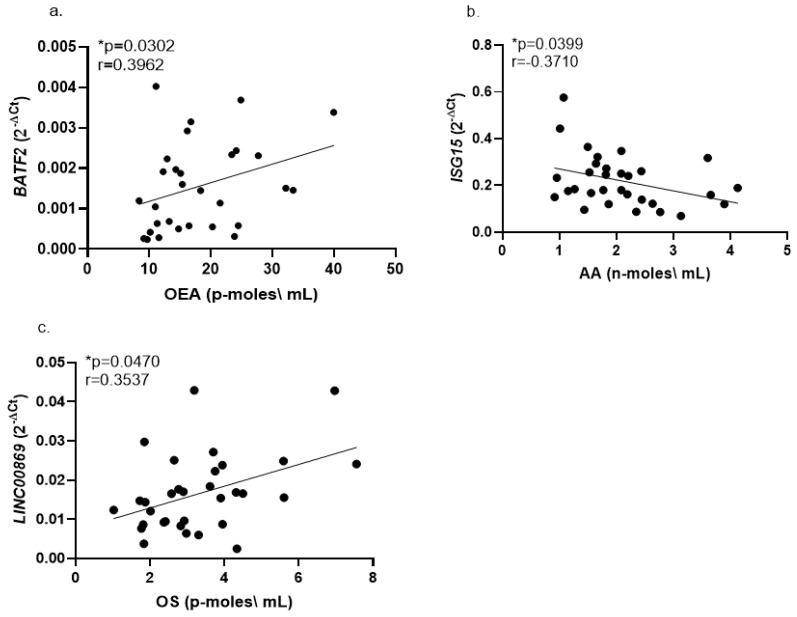
Correlations for whole blood mRNA expression levels with serum endocannabinoid levels in ASD children (Israeli cohort). Correlations are shown for (**a**) *BATF2* and oleoylethanolamide (OEA); (**b**) *ISG15* and arachidonic acid (AA); (**c**) LINC00869 and Oleoyl serine (OS). The r and *p* values for each correlation plot (Spearman test) are shown in each panel. Endocannabinoid levels are from Aran et al., 2019 [18]. * *p* < 0.05. See Section 4 for further details.

Reduced levels of several endocannabinoids were reported in serum samples of children with ASD compared with controls [18]. These serum samples were from the same Israeli ASD and NT children used in the current study for RNA analysis from whole blood samples. Therefore, in each of the study participants (both the ASD and NT control groups), we looked for correlations between blood RNA expression levels of the top dysregulated genes (Table 2) and each of the serum endocannabinoid levels reported by Aran et al., 2019 [18]. The significant correlations are shown in Figure 2. Further correlations for whole blood mRNA expression and serum endocannabinoid levels are listed in Supplementary Table S2. The strongest correlation observed was a negative correlation between whole blood *LY6E* mRNA expression levels and serum N-palmitoylethanolamine (PEA) in the NT control children (PEA; r = 0.7298, *p* = 0.0004), while no such correlation was observed for the ASD group (Supplementary Figure S3). Further correlations between blood mRNA expression levels and serum endocannabinoid levels were detected by combining the Israeli ASD and control groups for each correlation plot (Supplementary Figure S4).

## 3. Discussion

### 3.1. Upregulated BATF2 and LY6E and Downregulated ISG15 and MT2A Expression in Whole Blood of Children with ASD Compared with Neurotypical Children

Our RNA-seq of the Israeli blood samples from children with ASD compared with NT children identified 10 dysregulated genes following *p*-value correction for genome-wide analysis. Four of these ten genes were confirmed by our real-time qPCR analysis of the combined Israeli and American cohorts: upregulated *BATF2* and *LY6E* and downregulated *ISG15* and *MT2A* (Figure 1). Notably, the upregulated expression of *BATF2* and the downregulated expression of *ISG15* and *MT2A* were also observed when analyzing (qPCR) only the Israeli blood RNA samples; albeit, it did not validate the upregulated *LY6E* while detecting upregulated *FCGR1A* (Supplementary Figure S1). 

The upregulated expression levels of *BATF2* in our combined Israeli and American cohorts of whole blood samples from children with ASD (Figure 1) corroborate earlier findings from a Japanese RNA-seq study in whole blood from adults with ASD [19]. To our knowledge, our present study provides the first independent validation of a genome-wide RNA-seq study in blood samples from individuals with ASD. Our validation of upregulated *BATF2* in children with ASD, regardless of their ethnic background and age, supports the role of this gene product in the etiology of ASD. 

Our literature search of RNA-seq studies (Table 3) revealed a large variation in findings from earlier genome-wide transcriptomic studies (either RNA-seq or RNA microarray technologies) in whole blood samples of individuals with ASD compared to matched NT controls. Notably, with the exception of *FCGR1A* (validated only in our Israeli cohort; Supplementary Figure S1), the other dysregulated genes listed in Table 3 were each mentioned in only a single study (or a single meta-analysis [20]). Therefore, our current study appears to be the first validation of several dysregulated genes in blood samples from individuals with ASD. Moreover, our validation was performed on children with ASD (Table 1), in contrast to adults with ASD in the Japanese study [19]. 

Our RNA-seq detected *SERPING1* as the top upregulated gene in whole blood from children with ASD (FD = 3.4962; P_adj_ = 0.0072; Table 2). While we failed to validate *SERPING1* by qPCR of our combined cohorts, it was detected among the upregulated genes in the whole blood from Japanese adults with ASD [19]. Our findings of upregulated *FCGR1A* in the Israeli cohort (Supplementary Figure S1) constitute a validation of this gene as upregulated by two earlier genome-wide RNA studies [19,26] (Table 3). Our findings thus suggest a central role in ASD for the identified upregulated genes, independent of age and ethnicity. Further studies, including transcriptomic studies with brain tissues from ASD animal models, are needed to elucidate the implications of these dysregulated genes for ASD phenotypes.

### 3.2. Dysregulated ASD Genes and Cancer 

All four genes that were found dysregulated in the blood of children with ASD in our RNA-seq study and validated in our combined Israeli and American cohorts were previously studied mostly in the context of cancer. The protein coded by *BATF2* was shown to have an antitumor effect in a mouse model through upregulation of IL-12 p40 in tumor-associated macrophages, leading to CD8^+^ T-cell activation and tumor accumulation [27]. Among other cancers, BATF2 was demonstrated as a tumor suppressor of gastric cancer [28], glioblastoma [29], and esophageal squamous cell carcinoma [30]. In contrast, *LY6E*, the other gene found as upregulated in ASD blood samples, was suggested as a biomarker of poor prognosis in smoking-induced lung carcinogenesis [31] and colorectal cancer [32]. Notably, several conjugates of anti-LY6E antibodies were proposed as solid tumor therapeutics [33,34,35].

Higher mRNA expression levels of both *ISG15* and *MT2A*, found in this study as downregulated in blood samples from children with ASD, were reported to be associated with worse cancer prognosis. The protein coded by *ISG15* (interferon-stimulated gene 15 ubiquitin-like modifier) was implicated in autophagy, exosome secretion, DNA repair, and immune modulation pathways; it is also a known tumor promoter by suppressing immune cell tumor infiltration [36]. ISG15 was shown to drive tumorigenesis and metabolic plasticity of pancreatic cancer, suggesting that its inhibition may be a treatment option for pancreatic cancer [37]. Higher ISG15 expression was also associated with poor prognosis in breast cancer [38]. The protein coded by *MT2A*, metallothionein 2A, is the major metallothionein in humans, serves as a chelator of intracellular zinc ions, and protects cells against free radicals. MT2A is upregulated in most cancers and contributes to their chemotherapy resistance by chelation of zinc and platinum-containing drugs and by its action on p53 zinc-dependent activity. MT2A upregulation results in p53 misfolding secondary to zinc chelation, while low cellular MT2A levels allow proper p53 function as a genome stability guardian [39,40]. Lastly, downregulated C1 Inhibitor (encoded by *SERPING1*) was shown to increase cancer risk [41,42]. Hence, the upregulated *SERPING1* observed in our RNA-seq (while not validated in our combined cohorts) may also contribute to reduced cancer risk in children with ASD.

Taken together, the upregulation of *BATF2* and the downregulation of both *ISG15* and *MT2A,* as reported here in blood samples from children with ASD, suggesting a reduced risk of cancer. Indeed, a robust reduction in cancer risk (OR = 0.06; 95% CI: 0.02, 0.19; *p* < 0.0001) was reported among children with ASD aged from 0 to 14 years compared with matched controls [43]. These authors compared cancer rates in 1837 individuals with ASD and in 9336 controls in the registry of the University of Iowa Hospitals and Clinics. They observed that the large gap in cancer rates between individuals with ASD and controls was smaller at older ages, being only 2-fold less among individuals with ASD aged above 55 years compared with controls.

Our findings on upregulated *BATF2* and downregulated *ISG15* and *MT2A* in children with ASD thus seem to agree with the findings of the Darbro et al., 2016 [43] epidemiologic survey. Albeit, the upregulated *LY6E* in children with ASD might have cancer-promoting effects, thereby offsetting the anti-cancer effects of the other three dysregulated genes [31]. Of note, we did not identify similarly large studies on cancer risk among children with ASD. The only other epidemiologic study reporting reduced cancer risk among individuals with ASD was smaller (91 individuals with ASD and 6186 sex- and birth-year controls), and was based on death records, thus being less relevant for children. For all ages combined, it reported a 4.3-fold reduced risk of death from metastatic cancer in individuals with ASD compared with controls [44]. However, an earlier study reported a 1.95-fold higher cancer incidence among males with ASD based on a Taiwanese cancer registry; the elevated cancer risk was particularly high (3.58-fold) for individuals with ASD aged 15–19 years [45]. Additionally, higher cancer mortality (OR = 1.80) among individuals with ASD was reported in a study on premature mortality [46]. Yet, the latter study did not include a breakdown of death by age. Hence, the cancer risk among individuals with ASD remains controversial. Larger epidemiologic studies are required to assess the cancer risk among children with ASD compared with NT children.

### 3.3. Dysregulated ASD Genes and Immunity

Among the common phenotypic features observed in ASD is innate immune system dysregulation, leading to a chronic pro-inflammatory state [6,47]. The innate immune pathways affected in ASD include signaling mediated via cytokines, hepatocyte growth factor receptor, microglia, and the complement system. These suggest a role for aberrant immune function in the broad ASD phenotypes [48]. A recent RNA-seq study of whole blood from adults with ASD found dysregulated transcription of genes implicated in innate and adaptive immunity. These included upregulated expression of *BATF2* [19], as confirmed in our current study of whole blood from children with ASD compared to NT children. The consequences to the immune system of dysregulation of *BATF2*, *LY6E*, *ISG15* and *MT2A* in children with ASD (Figure 1) are discussed in the above section. Notably, BATF2 was shown to promote inflammation in response to lipopolysaccharides or infection [49], and LY6E plays a role in T-cell activation and is upregulated by interferons [50,51]. Additionally, ISG15 is known to promote anti-inflammatory pathways [52,53]. Thus, the upregulation of *BATF2* and *LY6E* mRNA, as well as the downregulation of *ISG15* and *MT2A* mRNA observed in our study, support the involvement of the pro-inflammatory phenotypes observed in ASD [6,7,47].

### 3.4. Correlations of Whole Blood Gene Expression Levels with Serum Endocannabinoids

Recent years have seen growing interest in studying the use of cannabinoid drugs for treating behavioral and social deficits of individuals with ASD [54]. The endocannabinoid system was reported to be dysregulated in various animal models of ASD [55]. Children with ASD were reported to have lower serum endocannabinoids [18], and rare mutations in endocannabinoid pathway genes were implicated in some persons with ASD [56]. Circulating endocannabinoids are derived from multiple tissues [57]. However, plasma endocannabinoid levels were demonstrated to reflect brain concentrations [58]. Hence, the correlations reported here for blood expression of some of the dysregulated genes with serum endocannabinoids (Figure 2) can shed light on the pathophysiology of ASD. However, studies in animal models of ASD, which allow measurements of endocannabinoid levels and transcriptomics in brain tissues during different stages of pre- and postnatal development [59,60], are required for exploring these correlations. The endocannabinoid PEA was reported to display robust anti-inflammatory [15,61], antiepileptic [62], and antineuropathic [63] properties. The LY6E cell surface protein was shown to be upregulated by several inflammatory cytokines, including interferons, TNF-alpha, and IL-1 alpha [50]. As expected, we found a strong negative correlation between the anti-inflammatory endocannabinoid PEA and the pro-inflammatory transcript *LY6E* in NT children (Supplementary Table S2, r = −0.73, *p* = 0.0004). This expected negative correlation was not observed in children with ASD, thus demonstrating another example of dysregulated immunity in ASD. Further studies are needed to clarify the relation of the dysregulated blood transcriptomics to the reduced serum endocannabinoids observed in children with ASD [18].

### 3.5. Study Limitations

Our current study has several limitations. First, the small cohorts do not enable separate analysis for males and females. As all the participants in the RNA-seq discovery cohort and the majority of the participants in both the Israeli and American cohorts were male (Table 1), the relevance of our findings to female children with ASD needs to be clarified. Future studies should therefore compare dysregulated genes in both male and female children with ASD. Second, the children with ASD in the American cohort were, on average, 5.8 years younger than those of the Israeli cohort (Table 1). Our lack of qPCR validation for six of the ten genes detected as dysregulated in our blood RNA-seq suggests that confirmations are required from larger cohorts. Additionally, dysregulated mRNA expression may differ from protein expression; future studies should therefore assess, in addition, the protein levels of the dysregulated mRNAs. Lastly, the relevance of our transcriptomic findings for early ASD diagnosis is uncertain, as only a few children in both the Israeli and the U.S. cohorts were under the age of four years, the most crucial period for early ASD diagnosis [12,13].

In light of the above reservations, we conclude that validation in larger cohorts, ideally including blood samples from both male and female younger children, is essential for assessing the potential diagnostic and prognostic values of the dysregulated genes detected in our current study. The implications for the upregulated blood transcription of *BATF2* and *LY6E* and downregulated transcription of *ISG15* and *MT2A* for the distinctive immune system phenotypes observed in individuals with autism, as well as the controversial published findings on cancer risk among children with ASD, merit further and larger studies.

## 4. Materials and Methods

### 4.1. Participants

The study comprised an Israeli and a U.S. cohort of children with ASD and matched NT controls. All experimental protocols were approved by the Shaare Zedek Medical Center Institutional Review Board (Israeli cohort) or the Phoenix Children Hospital Institutional Review Board (American cohort). The Israeli cohort was described by Aran et al., 2019 [18]. Briefly, children and young adolescents with ASD were recruited as part of a randomized clinical trial (NCT02956226) approved by the Shaare Zedek Medical Center Institutional Review Board (Jerusalem, Israel; IRB# 0501-20-SZMC, original approval 2 December 2020) and the Israeli Ministry of Health. Unrelated, age- and gender-matched, neurotypical (NT) children who attended regular education and were without any neuropsychiatric diagnosis other than attention deficit hyperactivity disorder were recruited through advertisements posted in the surrounding community of Jerusalem, Israel. The protocol for the American cohort is registered on clinicaltrials.gov as NCT04322734. This part of the study was performed and approved by the Institutional Review Board at Phoenix Children’s Hospital (Phoenix, AZ, USA; IRB# IRB-19-606, original approval 13 January 2020). For both the Israeli and the U.S. cohorts, the children’s parents provided written informed consent, and written assent was obtained from participants when appropriate. The study and its methods were performed in accordance with the approved protocols and the relevant guidelines and regulations.

### 4.2. Whole Blood Sample Collection

Whole blood samples were collected as described by Aran et al., 2019 [18]. Briefly, venous whole blood was collected in the morning hours from non-fasted individuals. Whole blood samples from the Israeli cohort were collected into Tempus^TM^ Blood RNA tubes (Applied Biosystems™ Catalog number 4342792, Thermo Fisher Scientific, Waltham, MA, USA). Whole blood samples from the American cohort were collected into PAXgene^®^ blood RNA tubes (Catalog number762165, BD Diagnostics, Franklin Lakes, NJ, USA), which were frozen immediately at minus 80 °C until RNA extractions were performed.

### 4.3. RNA Extraction

RNA was extracted using the Tempus™ Spin RNA Isolation Kit (Invitrogen™, Thermo Fisher Scientific, Waltham, MA, USA; Israeli cohort) or by the PAXgene^®^ Blood miRNA Kit (QIAGEN™, Germantown, MD, USA; American cohort) by following the manufacturers’ protocols. RNA quality was determined by an automated electrophoresis process using the TapeStation system (Agilent, Santa Clara, CA, USA). Samples with high-quality RNA were specified as those with RIN (RNA integrity number) >8. Nucleic acid quantitation was carried out by Qubit™ RNA HS Assay Kit (Invitrogen™, Thermo Fisher Scientific, Waltham, MA, USA). The samples selected for sequencing were diluted to 1 ug/mL.

### 4.4. Library Preparation, RNA Sequencing, and Data Processing

RNA samples were shipped in dry ice to Macrogen Europe BV (Amsterdam, The Netherlands) for poly-A mRNA sequencing. Libraries were prepared using a TrueSeq stranded total RNA LT sample prep kit (Illumina, San Diego, CA, USA). RNA sequencing was performed as a paired-end read on an Illumina True-Seq platform. Sequencing depth was ~30 M reads/sample. Raw sequencing data were trimmed using fastp 0.20.0 [64] and aligned to the GRCm38 assembly using STAR 2.6.0c [65]. DESeq2 1.30.1 [66] and R 3.6 were used for normalization of count data and for statistical analysis of differential gene expression in children with ASD compared with NT control children.

### 4.5. Real-Time qPCR Validation

Real-time quantitative PCR (qPCR) reactions were performed with 1 μg RNA samples converted to cDNA using qScript cDNA Synthesis Kit (Quanta Bio, Beverly, MA, USA). Reverse transcription was performed using a thermal cycler over three steps (22°C for 5 min, followed by 42 °C for 30 min and 85 °C for 5 min). Real-time PCR reactions were performed in mixtures containing 10 ng of cDNA, PerfeCTa SYBR^®^ Green FastMix Kit (Quanta Bio, Beverly, MA, USA), and Integrated DNA Technologies, Inc. (Leuven, Belgium) primers. *GAPDH* (Glyceraldehyde 3-phosphate dehydrogenase) was used as a reference gene. Forward and reverse primer sequences for RT–qPCR are listed in Supplementary Table S1.

### 4.6. Correlations and Statistical Analysis

Real-time qPCR data analysis was conducted using GraphPad Prism v.9 (San Diego, CA, USA) software. The normality of the data distribution was evaluated using the Shapiro–Wilk test; continuous variables between two groups were analyzed by the Mann–Whitney test; outliers were detected by the ROUT test. *p*-values ≤ 0.05 were considered significant. Correlations were examined by assessing the normality of data distribution using the Shapiro–Wilk test, followed by a Spearman correlation test.

### 4.7. Behavioral Tests

Behavioral tests for the participants of the Israeli cohort were described by Aran et al., 2019 [18]. Briefly, they included the Vineland Adaptive Behavior Scale Composite and socialization domain scores (VABS), the Social Responsiveness Scale (SRS), and the Child Behavior Checklist (CBCL). Behavioral tests for the participants of the U.S. cohort included, in addition, scores on the Aberrant Behavior Checklist (ABC).

### 4.8. Literature Survey of Transcriptomic Studies in Whole Blood from Individuals with ASD and Controls

We searched the NCBI PubMed database for studies published from January 2010 to June 2022 describing transcriptomics of whole blood samples from individuals with ASD compared to controls. Inclusion criteria were the following words in the abstract: transcriptomics, RNA-sequencing (or RNA-seq), or microarrays; and also both words autism and blood. RNA-seq studies with other biological human samples (such as postmortem tissues) or with fewer than six blood samples were excluded.

## Figures and Tables

**Table 1:** Israeli and U.S. cohorts of whole blood samples from children with autism spectrum disorder (ASD) and neurotypical children (control). Differences in mean age between the groups within each cohort were not significant (*p* > 0.1). The mean ages of the ASD and NT children were lower in the U.S. than in the Israeli cohort (*p* < 0.0001 and *p* = 0.0056, respectively). * For the U.S. cohort, these behavioral scores were not available for a minority of the children with ASD. VABS: Vineland Adaptive Behavior Scale; SRS: Social Responsiveness Scale; CBCL: Child Behavior Checklist; ABC: Aberrant Behavior Checklist.

	*Israeli Cohort*		*U.S. Cohort*	
	Neurotypical controls	Children with ASD	Neurotypical controls	Children with ASD
*N*	21	36	5	37
*Age (years)*	16.3 ± 2.7	13.2 ± 4.8	8.9 ± 5.4	7.4 ± 3.7
*% Male*	75%	83%	76%	84%
	*Behavioral scores*			
*VABS*	N/A	46 ± 11	N/A	N/A
SRS *	N/A	78 ± 10	N/A	78 ± 13
*CBCL*	N/A	55 ± 11	N/A	N/A
ABC *	N/A	N/A	21 ± 29	60 ± 22

**Table 2:** Top transcripts showing differential expression in whole blood RNA sequencing. RNA-seq reads are shown for children with autism spectrum disorder (ASD; N = 8) vs. neurotypically developing children (control; N = 9) with adjusted Padj < 0.05. Padj and *p*.-values were obtained using DESeq2 (see Methods). The genes are arranged by increasing adjusted *p* values (P_adj_). FD, fold difference (ASD vs. controls). Gene names in bold fonts indicate the genes validated as dysregulated by real-time qPCR in the combined Israeli and American cohorts.

*Gene Name*	*Gene ID*	*Mean Reads*	*FD*	*p-Value*	*P_adj_*
*SERPING1*	ENSG00000149131	494.495	3.4962	3.73 × 10^−7^	0.0072
*EFHC2*	ENSG00000183690	93.312	0.6086	2.81 × 10^−6^	0.0261
***BATF2***	ENSG00000168062	127.616	3.4360	4.06 × 10^−6^	0.0261
*CDC20*	ENSG00000117399	66.744	3.1642	6.06 × 10^−6^	0.0292
*FCGR1A*	ENSG00000150337	416.310	2.6044	1.37 × 10^−5^	0.0379
***MT2A***	ENSG00000125148	407.810	1.8252	1.35 × 10^−5^	0.0379
***ISG15***	ENSG00000187608	1262.341	3.1128	1.36 × 10^−5^	0.0379
*FBXO6*	ENSG00000116663	365.625	1.5121	1.89 × 10^−5^	0.0416
*LINC00869*	ENSG00000277147	306.080	1.4388	1.94 × 10^−5^	0.0416
***LY6E***	ENSG00000160932	3225.314	1.8098	2.49 × 10^−5^	0.0481

**Table 3:** Transcriptomic studies in whole blood samples from individuals with autism spectrum disorder (ASD) and NT controls. The NCBI PubMed database was searched (July 2022) as described under Methods. The abbreviations of dysregulated genes showing differential expression with P_adj_ < 0.05 are presented. Studies are shown chronologically for each technology. (a) RNA-seq studies; (b) meta-analysis of blood-based microarray data sets. Genes found as dysregulated in ASD in our study (Table 2) are indicated by bold fonts.

Study	Dysregulated Genes
a. RNA-seq studies	
Walker et al., 2016 [21]	*CYP2S1, TNFRSF12A, IL1RN, TNFAIP3, SIGLECP3*
Saffari et al., 2019 [22]	*IGHG4, PRR13P5, DEPDC1B, ZNF501, EVI2A, SNORD15B*
Filosiet al., 2020 [23]	*NMUR1, HMGB3, PTPRN2*
Horiuchi et al., 2021 [19]	*ETV7, **BATF2**, **SERPING1**, CD274, **FCGR1A**, WARS, CASP5, PI3, RPSAP58, GZMK, KLRB1, GZMA, DUSP2, GNLY, A2M-AS1, TOMM7, LINC00612, SNORD3A*, *FCGR1B, TMEM176B*
**b. Meta-analysis of blood-based microarray data sets**	
Gregg et al., 2007 [24]	*PAM, SPON2, IL2RB, PRF1, GZMB, CX3CR1, SH2D1B/EAT*
Kong et al., 2012 [25]	*CREBZF, HNRNPA2B1, KIDINS220, LBR, MED23, ZDHHC17, ZMAT1, ZNF12, SULF2, RBBP6, SPATA13, TMEM30A*
Glatt et al., 2012 [26]	***FCGR1A*** *, ANKRD22, GBP5, L-RAP, FCGR1B, GBP1, LGALS2, ANXA3, LILRA3, ADM, PLSCR1, NAMPT, HLA-H, STX11, PTER, BC050625, SLC22A4, IL1RN, BTN3A2, S100A12, SLC26A8, GNG10, DQ893795, USP10, PARP9, CST7, GCH1*
Lee et al., 2019 [20]	*TTF2, UTY, KCNJ8, NCS1*

## Data Availability

The datasets generated during the current study, including RNA-seq datasets, are available from D.G. on reasonable request.

## Supplementary Materials

The following supporting information can be downloaded at: https://www.mdpi.com/article/10.3390/ijms23179843/s1.
