# Supplementary material for: Blood RNA Sequencing Indicates Upregulated BATF2 and LY6E and Downregulated ISG15 and MT2A Expression in Children with Autism Spectrum Disorder
Source: Int J Mol Sci. 2022 Aug 30;23(17):9843. doi: 10.3390/ijms23179843 (PMC9456089; doi:10.3390/ijms23179843)

Supplementary Tables and Figures for:

Blood RNA sequencing indicates upregulated *BATF2* and *LY6E* and downregulated *ISG15* and *MT2A* expression in children with autism spectrum disorder

Irena Voinsky, Yazeed Zoabi, Noam Shomron, Moria Harel, Hanoch Cassuto, Joseph Tam, Shannon Rose, Adrienne C. Scheck, Mohammad A. Karim, Richard E. Frye, Adi Aran, and David Gurwitz

Supplementary Table S1: **Real-time qPCR primers used for real-time qPCR.**

Gene	*Forward*	*Reverse*
GAPDH	GGAGCGAGATCCCTCCAAAAT	GGCTGTTGTCATACTTCTCATGG
SERPING1	CCTTGTCCTCCTCAATGCTATC	CCACAGGGTACTTCTTGCTATT
EFHC2	ATGGCCGGACATTCAAGATTT	GGCTCTACGTGTTCTACAACCT
BATF2	CCTCATTGCTCTTTGGACTAGG	TGTGTGAAACCTGGGTGAAG
CDC20	AGACCTGCCGTTACATTCCTTC	GCCAGTACATTCCCAGAACTCC
FCGR1A	CGCTACACATCAGCAGGAATA	GCCCATGTAGAAGGAGAAGTAAA
MT2A	CCGCTCCCAGATGTAAAGAA	CACGGTCAGGGTTGTACATAA
ISG15	CGCAGATCACCCAGAAGATCG	TTCGTCGCATTTGTCCACCA
FBXO6	ATCCTACGAAATGTGCCTCAAG	CCAACACGAAGTAGTCAGCCG
LINC00869	CATCCAAAGCTACTGCCTACTC	CACCTCTCCCTTCCTGTATCT
LY6E	GGGAATCTCGTGACATTTGGC	ACACCAACATTGACGCCTTCT

Supplementary Table S2: Summary of Spearman correlation test of top 10 RNA-seq genes and serum endocannabinoids. (a) ASD samples only; (b) neurotypical controls; (c) ASD and neurotypical controls combined. Outlier samples were removed. P-value is two-tailed; N, XY pairs. Serum endocannabinoid levels are taken from Aran et al. 2019. Correlations with p<0.05 are shown in bold fonts.

**A. ASD**		*SERPING1*	*EFHC2*	*BATF2*	*CDC20*	*FCGR1A*	*MT2A*	*ISG15*	*FBXO6*	*LINC00869*	*LY6E*
	N (XY Pairs)	22	33	30	25	29	31	31	28	32	30
**OEA**	p.val	0.4166	0.9617	**0.0302**	0.3610	0.7452	0.2111	0.7679	0.7148	0.2676	0.8491
	r	0.1824	-0.00869	0.3962	0.1908	0.06305	0.231	0.05524	0.07225	0.202	0.03626
**PEA**	p.val	0.0637	0.9199	0.8702	0.5284	0.7093	0.7767	0.9764	0.4859	0.0779	0.2635
	r	-0.01822	0.05131	-0.06855	-0.1323	0.005348	0.1257	0.3162	0.2004	-0.01822	-0.06855
**AEA**	p.val	0.64	0.1019	0.2843	0.6634	0.7127	0.2963	0.816	0.3895	0.1845	0.3628
	r	-0.1056	-0.2898	0.202	-0.09154	0.07144	0.1938	-0.04355	-0.1692	-0.2407	-0.1722
**OS**	p.val	0.1571	0.4824	0.3038	0.8096	0.8451	0.1035	0.4987	0.6617	**0.047**	0.2331
	r	0.3123	0.1267	0.1942	0.05077	-0.03793	0.298	-0.1262	0.08648	0.3537	0.2245
**2AG**	p.val	0.4934	0.7297	0.7516	0.4038	0.5758	0.3308	0.4973	0.9098	0.296	0.1326
	r	0.1542	-0.0625	-0.06029	-0.1746	0.1084	-0.1806	0.1266	-0.02244	0.1906	0.281
**AA**	p.val	0.9106	0.1811	0.973	0.5828	0.9697	0.0718	**0.0399**	0.083	0.645	0.2182
	r	-0.02541	-0.2386	-0.00645	-0.1154	0.007389	0.3278	-0.371	-0.3333	-0.08468	0.2316

**B. NT controls**		***SERPING1***	***EFHC2***	***BATF2***	***CDC20***	***FCGR1A***	***MT2A***	***ISG15***	***FBXO6***	***LINC00869***	***LY6E***
	N (XY Pairs)	18	19	18	17	17	19	16	19	18	18
**OEA**	p.val	0.1881	0.4680	0.6042	0.2311	0.9887	0.7372	0.0540	0.5092	0.9384	0.8357
	r	-0.3251	0.1772	-0.1311	-0.3064	-0.00490	0.08246	-0.4941	-0.1614	0.01961	-0.05263
**PEA**	p.val	0.9659	0.8700	0.5764	0.1842	0.0617	0.9398	0.7802	0.6045	0.7264	**0.0004**
	r	0.01053	0.03910	-0.1368	-0.3382	-0.4489	-0.01805	-0.07353	0.1233	0.08596	-0.7298
**AEA**	p.val	0.4415	0.9248	0.3515	0.1156	0.8869	0.7865	0.1619	0.349	0.3553	0.6576
	r	-0.1877	-0.02256	-0.2263	-0.3971	0.03612	0.06466	-0.3554	0.2211	0.2246	-0.1088
**OS**	p.val	0.9205	0.4237	0.7753	0.3568	0.7851	0.3907	0.2853	0.7005	0.6785	0.4907
	r	-0.02456	0.1895	-0.07018	-0.2377	0.06914	0.203	-0.2745	-0.09173	0.1018	-0.1684
**2AG**	p.val	0.1408	0.1408	0.5617	0.8835	0.7048	0.5395	**0.0137**	0.4778	0.101	0.9602
	r	-0.3509	0.3414	0.1421	-0.03922	0.09598	-0.1459	-0.5931	0.1684	0.3877	-0.01228
**AA**	p.val	0.2206	0.7527	0.2797	0.8686	0.2875	0.2272	0.6322	0.1838	0.9488	0.5375
	r	0.2947	-0.07519	-0.2614	-0.04412	-0.2652	0.2827	-0.125	-0.3098	-0.01579	0.1509

**C. ASD and NT controls**		***SERPING1***	***EFHC2***	***BATF2***	***CDC20***	***FCGR1A***	***MT2A***	***ISG15***	***FBXO6***	***LINC00869***	***LY6E***
	N (XY Pairs)	34	51	49	42	48	51	47	48	52	49
**OEA**	p.val	0.8055	0.8063	0.6085	0.8773	0.4609	0.0957	0.8464	0.7617	0.2714	0.384
	r	0.04385	-0.0352	0.075	-0.02455	-0.109	0.2358	-0.02902	0.04494	0.1554	-0.1271
**PEA**	p.val	0.24	0.8426	0.5492	0.1955	**0.0098**	0.152	0.1997	0.5513	0.2708	**0.0216**
	r	0.207	-0.02851	-0.08766	-0.2038	-0.3693	0.2035	0.1905	0.08816	0.1556	-0.3275
**AEA**	p.val	0.5675	0.1005	0.5462	0.2127	0.2017	0.1744	0.9186	0.9259	0.7398	**0.0481**
	r	-0.1016	-0.2326	-0.08832	-0.1963	-0.1876	0.1932	-0.01532	-0.01379	-0.04717	-0.2839
**OS**	p.val	0.1573	0.8367	0.3639	0.9647	0.9271	0.1147	0.1111	0.7004	**0.0471**	0.6837
	r	0.248	0.02959	0.1326	-0.00705	0.01357	0.2236	-0.2354	0.05699	0.2767	0.05969
**2AG**	p.val	0.6372	0.9362	0.3366	0.4241	0.3184	0.0741	0.4127	0.9947	0.0571	0.1239
	r	0.08388	-0.01149	0.1402	-0.1267	0.1471	-0.2523	-0.1223	0.000977	0.2655	0.2228
**AA**	p.val	0.5781	0.1857	0.8734	0.6996	0.7157	0.0708	**0.0314**	**0.0242**	0.6809	0.0995
	r	0.09885	-0.1883	-0.02337	-0.06134	-0.05395	0.2551	-0.3143	-0.325	-0.0584	0.2381

Supplementary Figure S1: Real-time qPCR validation for whole blood RNA expression levels in ASD and control children from the Israeli cohort alone. Box plots show mean ± SEM mRNA levels for ASD vs. neurotypical control whole blood samples. Outliers were removed and analysis was done using a non-parametric Mann Whitney test. FD and p values are shown for the genes with differential expression in ASD vs. neurotypical controls.

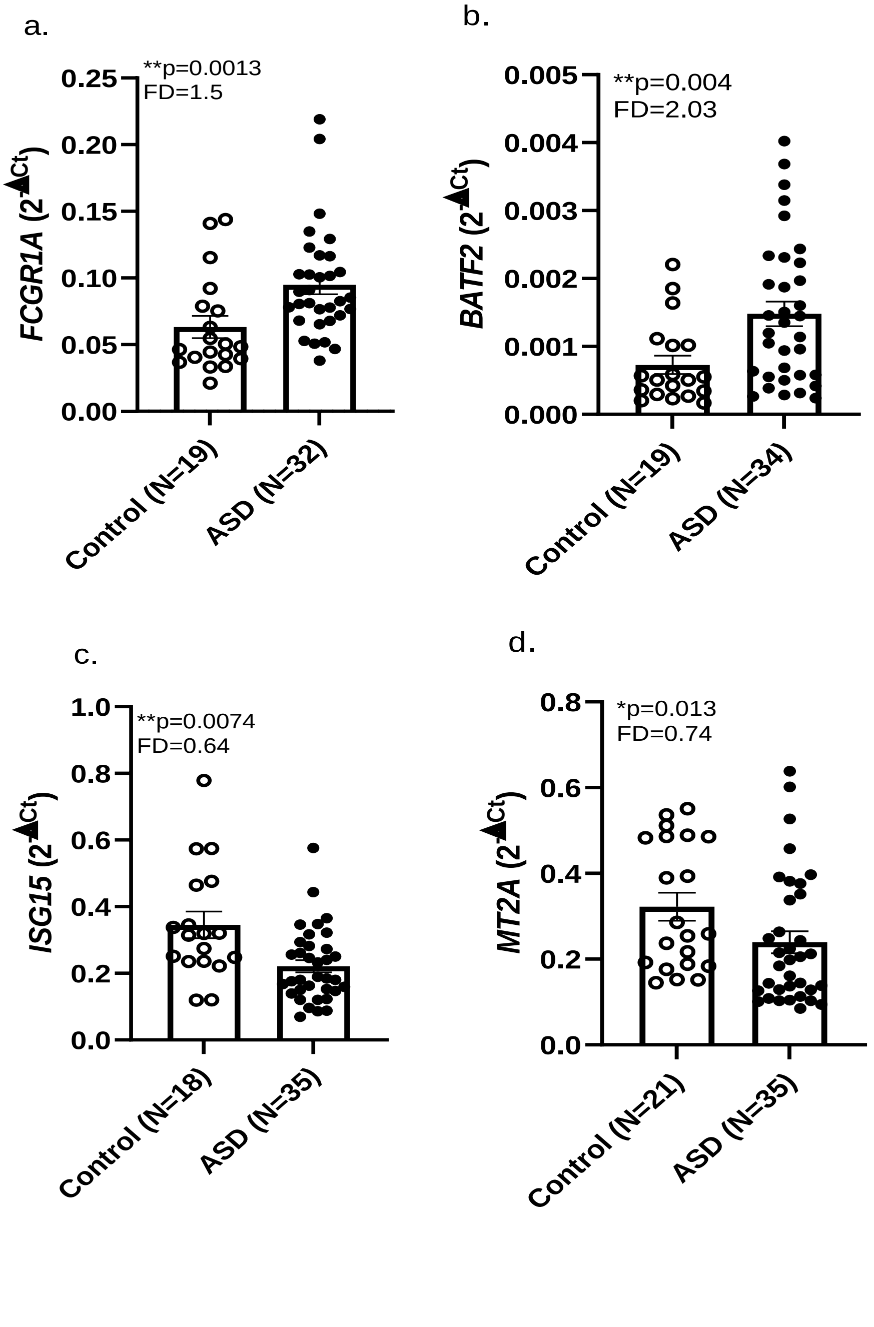

Supplementary Figure S2: Whole blood mRNA expression levels of *BATF2*, *LY6E* and *ISG15* correlate with ASD behavioral scores (Israeli cohort). Correlations for mRNA levels (real-time Pare shown for (a) *BATF2* vs. tSRS score (N=27); (b) *LY6E* vs. VABS socialization domain score (N=32); (c) *LY6E* vs. VABS Composite score (N=32); (d) *ISG15* vs. CBCL score (N=33). The r and p values for each correlation plot (Spearman test) are shown in each panel. See Methods for further information on behavioral scores.

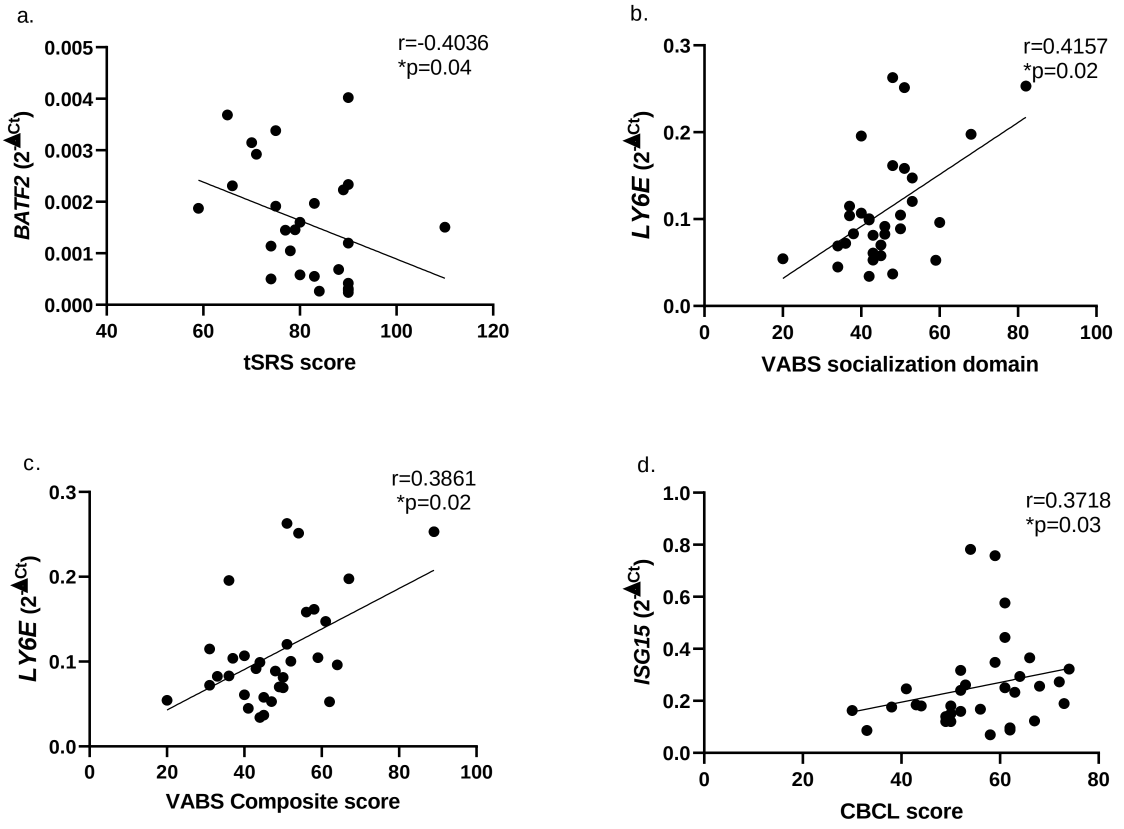

Supplementary Figure S3: Correlations for whole blood *LY6E* expression levels with serum palmitoylethanolamide (PEA) levels in children with ASD and neurotypical controls (Israeli cohort). Correlations are shown for (a) neurotypical control children (N=19); (b) ASD children (N=30). The r and p values for each correlation plot (Spearman test) are shown in each panel. PEA levels were taken from Aran *et al.* 2019. See Methods for further details.

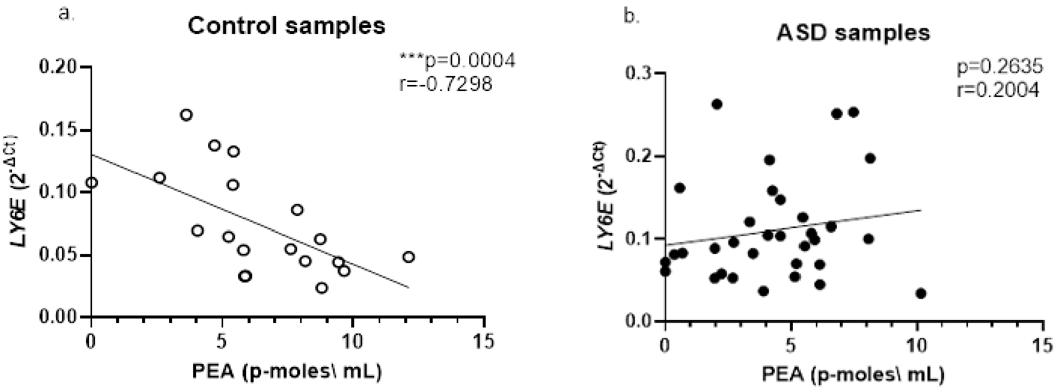

Supplementary Figure S4: **Correlations for whole blood mRNA expression levels with serum endocannabinoid levels in children with ASD and neurotypical controls combined (Israeli cohort).** Correlations are shown for (a) *FCRG1A* and palmitoylethanolamide (PEA); (b) LY6E and PEA; (c) *FBXO6* and arachidonic acid (AA); (d) *ISG15* and AA*;* (e) LINC00869 and oleoyl serine (OS); (f) *LY6E* and anandamide (AEA). The r and p values for each correlation plot (Spearman test) are shown in each panel. Open circles indicate control children, while closed circles indicate ASD children; individual findings for whole blood mRNA expression and serum EC levels were combined for controls (open circles) and children with ASD (closed circles) for calculating r and p values for each correlation. Endocannabinoid levels were taken from Aran *et al.* 2019.

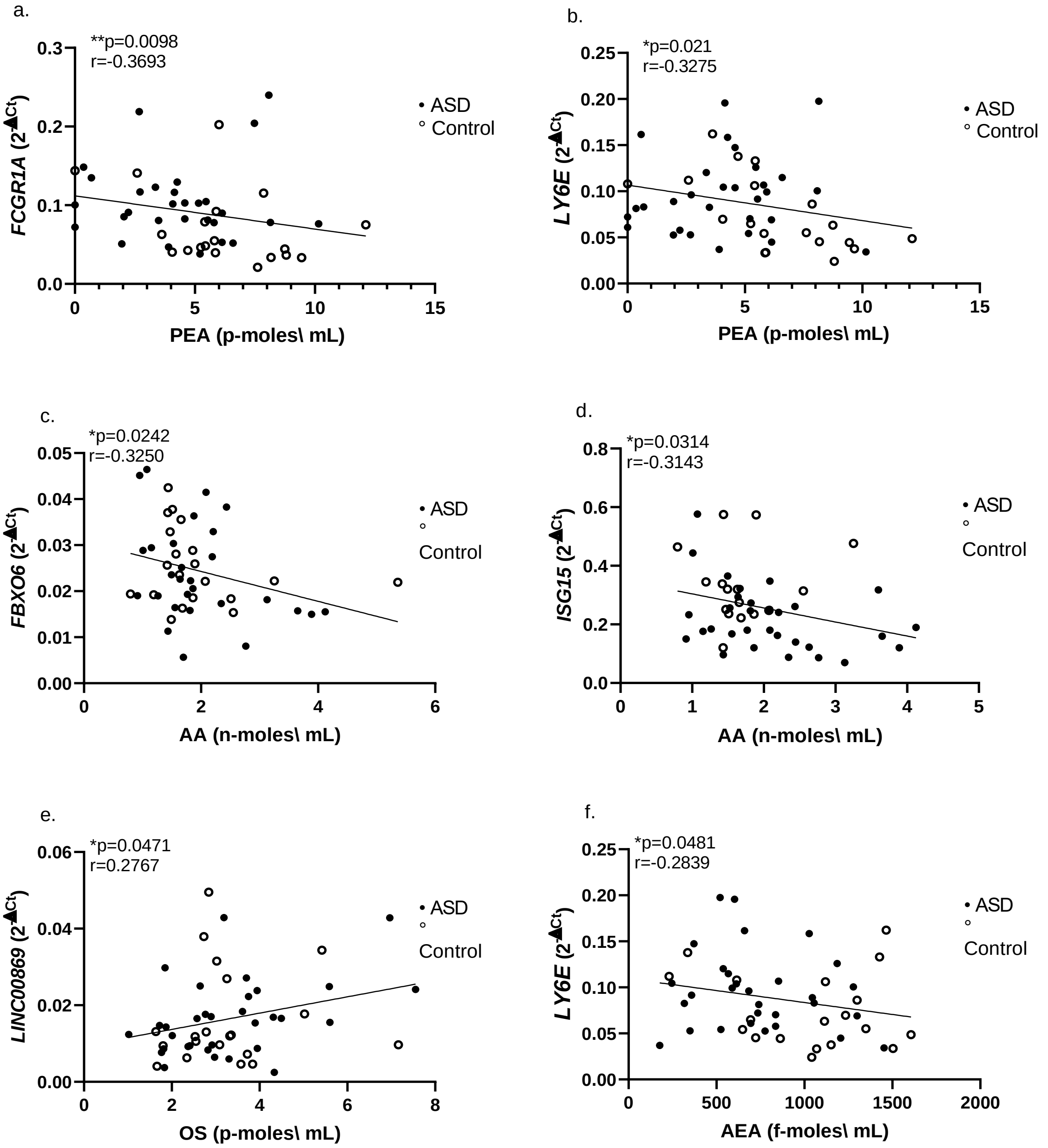

Supplement: Supplementary file 1 [file ijms-23-09843-s001.zip › ijms-1881799-supplementary.pdf]
